# Extra Supplement—The Nursing Mirror

**Published:** 1890-10-25

**Authors:** 


### "The Hospital" Nursing Mirror.

Being the Extra Nursing Supplement of "The Hospital" Newspaper.

All Contributions for this Supplement should be addressed to the Editor, The Hospital, 140, Strand, London, W.C., and should have the word "Nursing" plainly written in left-hand top corner of the envelope.

### En Passant.

WATFORD NURSES.—We have received the seventh report of the District Nurses for Watford. It is an unpretentious little pamphlet, but tells of real good work: 179 cases nursed by a staff of two—Miss Scott and Mrs Godfrey. Miss Scott is highly praised by the committee. The income last year was £201, and we are glad to see the society is not in debt, though it needs more funds to support a third nurse.

SERVANTS OF THE POOR.—Amongst the many schemes of district nursing in London is that carried out by the Sisters-Servants of the Sacred Heart of Jesus, Hassell Road, Homerton. Their principal work is visiting and nursing the sick poor in their own homes; they attend to the poor of all denominations, and they work gratuitously. The advantage these Sisters have over other systems is elasticity; they will attend contagious cases, they will sit up all night, they will wash the children and end the patient's clothes, they are, in fact, what they profess to be, "Sisters-Servants." On the other side, they openly state that their object is to minister to the souls as well as the bodies of their patients, and particularly to procure for them spiritual assistance in their last hours.

ANOTHER NICE STORY.—Here is another newspaper cutting, telling of an inquest held at Ealing, the particulars of which should uphold the Midwives' Registration Bill: "Mrs. Lamont Jackson, widow, said that she nursed Mrs. Comber in her confinement. The child was found dead on Tuesday morning, at twenty minutes to seven o'clock. She was quite sure it was Tuesday. Perhaps she had made a mistake; it might have been Wednesday. The child was not lying between herself and the mother, but on the mother's breast. She had last attended to it two hours previously, when she gave it some milk and water. It was fretful then." The two women and the child were evidently in the same bed. And while we are addressing midwives we would just call their attention to a simple combination for breast relieving. The ordinary breast-reliever is attached to a common glass syringe by an india-rubber tube; the syringe is then worked with the short intermittent action of a child sucking. The method is cleanly, rapid, and less painful than the ordinary method.

SHORT ITEMS.—Miss Dorothy Dix was the head of the American nurses during the Civil War: she is still alive.—The German Congress of midwives has decided to petition the Empress and the Government to alter the official designation of a midwife from "Hebamme" to "Geburtshelferin."—Sister Rose Gertrude is expected back to Europe shortly.—The current number the *Englishwoman's Review* contains a pathetic story of one of the Sœurs de Bon Secours de Troyes. This nursing heroine defended a small patient from a mad dog, was herself bitten very badly, and subsequently died of hydrophobia.—Nurses Agnes and Eva of St. John's Hospital, Twickenham, have had their salaries raised as a mark of appreciation of their devoted service.—Miss Winifred Sweet, a lady journalist, has visited the leper settlement at Molokai, and is the only woman who has done so, save the Sisters of Mercy.—The Home of Rest for Ladies at Pau, under Miss C. Watson, re-opens this month.—Three private nurses write to propose a badge to distinguish trained nurses from domestic servants. Such a snobbish notion could only have occurred to those sadly in need of a badge to denote their standing in Society.

A NURSES' HOTEL.—When we were busily engaged in trying to house many of the nurses who came to town for the Marlborough House function last summer, a ready response to our needs came from Miss Culverhouse, of 18, Royal Avenue, Chelsea. So pleased were some of the nurses with the accommodation and welcome they found there, that they returned again and again to the house, till two of them have come to regard the place almost as their home. Quite unexpectedly Miss Culverhouse finds herself keeping what is practically an hotel or boarding-house for nurses, and, what is more, the occupation proves pleasing; and so Miss Culverhouse means to continue it. To make the home as useful as possible, Miss Culverhouse is going to keep a register of the qualifications and terms of the nurses residing with her, for doctors of the neighbourhood have already learnt that they can generally pick up a nurse at 18, Royal Avenue, if they want one in a hurry. The charges are moderate, something like 1s. 6d. for a bed for the night, and other things in proportion. Private nurses living in the neighbourhood of Royal Avenue might with advantage call on Miss Culverhouse.

NORTHERN WORKHOUSE INFIRMARIES.—At the Poor Law Conference at Southport, Dr. Rhodes read a paper in which he advocated the formation of a society for the supply of trained nurses to Workhouse Infirmaries for the North of England, on the lines of that already existing in London, for the promotion of this most desirable reform. It is hardly necessary to insist upon the advantage of employing trained nurses in place of the utterly inefficient and often morally degraded pauper attendants who were once the only persons to minister to the wants of the sick poor. What is wanted is an extension of the practice until the pauper nurse becomes as extinct as the dodo. It is fitting, too, that a fresh move in the direction of reform should be made in Lancashire, where the memory of what Agnes Jones did for the Liverpool workhouse infirmary is still fresh. In the course of the discussion of the subject, Miss Wilson answered questions and gave various explanations with reference to the work of the London Association. She said that since the Association came into existence in 1879 it had trained 143 nurses. Their income averaged only £250, so that the number they could train was limited. They trained some nurses at their own expense, and they received others who had come to them from recognised institutions and into whose character they inquired. They were very careful in seeing not only to the ability but to the good character of all nurses sent out. Before any nurse was sent out a certain salary must be promised. No nurse could be sent out under £20 a-year salary, with uniform and all found. It was not found possible to get good nurses at a lower salary. The boards of guardians which subscribed to the Association gave exactly what they pleased. Some gave a guinea and others seven guineas. Northern women would take better with northern patients, and on that account she thought it was very desirable an association should be formed for the northern counties. The President said he was sure they were all much obliged to Miss Wilson for her explanations, and he hoped the association would be formed. Lady Edward Cavendish, who had hoped to be present, had promised to assist in the work. The following gentlemen were appointed to confer with the ladies with reference to the proposed association, viz.: Dr. Rhodes (Chorlton), Mr. A. M'Dougall, jun. (Manchester), Mr. Richmond (Ormskirk), Mr. H. J. Hagger (Liverpool), and Mr. J. Whittaker (Burnley). It is hoped that the north will support its branch more generously than is done in the south.

### Practical Hints for Nurses.

In the course of a long nursing experience I have found out for myself the following small points, and may, perhaps, save some of my sister workers some trouble by stating them.

Reflector.—In private nursing, where a surgical lamp is not to be had, and an examination of a throat, or some other part, is going on, a spoon tied to a candle, so that the bowl of the spoon is just behind the flame, will be found very useful.

Spittoons.—In district nursing, if using a tea-cup or soap-dish for this purpose, line it with paper, and see that the paper is burnt. This saves much trouble.

Sling.—If a man injures his arm you can, with three safety-pins, so pin the sleeve to the coat as to make a perfect sling.

Respiration.—Never let a patient know you are counting the respirations. Remember that in an infant the respiration is 50 to a minute, in a child 36 to a minute, in a grown-up person 16 or 18 to a minute. If the respiration of an adult is over 20 a minute it should always be recorded, though it may rise as high as 40 a minute. Always provide yourself with a watch with a minute hand. If your patient is conscious, count the respirations by sight; if unconscious, by laying one hand gently on the chest. If possible, count the respirations when the patient is asleep.

Pulse.—Always count the pulse more than once, and strike an average. If you are a district nurse, you will find that at the commencement of your visit the pulse will probably be five or six beats higher than at the close. Remember, that in infants the pulse is 120 to a minute; in children, 70 to 80; in adults, 60; in the aged, 50. Here, again, the necessity of a watch with a minute hand is evident. For myself, I commenced my nursing career with a pretty little gold watch with erratic tendencies; but I soon changed it for a sensible silver watch with a white face, easily seen, and a minute hand.

Diagrams.—It is a great pity our nursing manuals are not better illustrated. I should advise every nurse to draw a diagram of the circulation of the blood, and colour it red and purple if she desires to understand the subject thoroughly. She can then paste it into her favourite nursing manual. A diagram of the thoracic and abdominal regions should also be drawn, for often you get the order to put a poultice to the left iliac or right lumbar region and are not at all sure where these lie. A sketch of a skeleton, with the arteries put in in red, and all the bones named, should also be drawn. Probationers in hospital can always get medical books from the library from which they can copy these diagrams. Any word or abbreviation met with and not known should be 'made a note of,' looked up, and the meaning entered on the margin or spare pages of the nursing manual. In this way a nurse gradually comes to have one book to carry about with her in which is all she needs to know. I have a copy of Miss Lückes' "Lecture to Nurses," but all the advertisement and title pages are covered with notes and diagrams, and I would not part with my copy for worlds.

Case-taking.—Always take private notes, for your own use, of all your cases. Not long dissertations, but just notes of temperature, pulse, respiration, and treatment. If you attempt a full account of every case you will probably end with a complete account of none. It is a great help to be able to look back and see how previous cases got on, and what complications ensued in a certain disease, and how long was the convalescence. When I am too tired to read I often pick up my little case-book and study its record—so mystic to outsiders, so full of meaning to me!

Surgical Instruments.—The very first day I was in a hospital ward the sister sent me to fetch an ophthalmoscope. I, not having the least idea what that instrument was like, was delighted to find in the surgical cupboard a box so labelled. But to learn the names of all the surgical instruments is a very difficult proceeding, which I got over in a very happy manner. I was buying new forceps at an instrument maker's, when he showed me his illustrated catalogue of instruments. I asked if I might have a copy, and he promptly presented me with a last year's list, stating that he charged for the new list, but would gladly give me the old. I have found that catalogue invaluable.

### Six Months in a Hospital.

By a "Special Pro."

(*Continued from page* xi.)

### IV.

I have just returned from my two hours off duty (four to six to-day), during which I went to the Birkbeck to see the results of the science and art examinations. My name was down for first class elementary. So glad. I hate being second! My day duty is in the female surgical ward, and my evening ditto is also feminine, but quite different, viz., obstetric. One of our medical students, a charming girl, and I have been sitting by the fire, nursing twins, both of whom died. The mother seemed very glad. I presume this is the effect of poverty upon the human constitution. From my point of view I also rejoice, but that is a different one from the other. Only last night a man was brought in, who had been taking laudanum to put an end to his life. He was marched round and round our square for two hours by the policeman who brought him, and had three pints of black coffee to drink, and they got him over it. I don't see why a poor fellow should not be allowed to die comfortably if he does it intentionally, and knows how to go about it! Of course you will be very shocked by my sentiments. As this ward is usually a very quiet little place in the evening, the nurse in charge has permission to go into the next, which is our chief accident ward, and help there if necessary. I went in last night to help, and found a "railway smash," as we call it. The poor fellow died after being in an hour. They are holding a post-mortem on him to-night. He had fractured his spine and ribs, and ruptured liver and lungs as well. The hæmorrhage was past stopping; it literally poured out of two tiny wounds in the back not bigger than a large pin's head. All we could do was to put a large pad of cotton-wool under it.

To-day I have been extremely busy. One patient died of bronchitis; a new case came in; five sick babies and a small child that persistently cries to go home, &c. This is a specimen baby—diet to be given every hour, 1 oz. milk, 1 oz. barley water, 20 drops of brandy, 2 drachms of lime water, all mixed together.

I have a dear little fellow, four years old, with a very slight attack of measles, and as we can't get rid of him—the infirmary, &c., refusing to take him—we have been obliged to put up with him. It was a case of either keeping him here or sending him home to one room with three or four brothers and sisters; so the doctors charitably resolved to let him stay. He has had very little the matter with him, and will be put in a general ward in a day or two. If it were not that there is a general ward on the same floor with the "isolated" it would be perfectly solitary confinement, not for fear of infection, but because there is no nurse to "relieve" in this ward, so I have to stay on duty from 9 a.m. till 9 p.m.; meals and all, here. Yesterday we had quite a lively time up here. A man suffering from pneumonia went "off his head," as they often do, and tried to go out. The ward nurse took his out-door clothes away, and put them upon a shelf in the bath-room, but he forced his way through the door to get them. She caught hold of them and threw them to me, and I simply tore out of the ward with them as fast as I could to get them out of sight. Then he said he would go home, whether he had them or not, and got to the door; and there was Nurse A—— pulling on one side to keep it shut, and shouting to me to run for a porter, and he, on the other side, pulling with all his might to open it. I flew downstairs and across the square and fetched a man up, expecting to find Nurse A—— on the floor when I got back. He quieted down, however, a little, and as he positively insisted on going, he was finally "discharged at his own request." These nurses do get courageous, no mistake. He was so strong, that when they tied him in bed with bandages, he let them do it quite quietly, and when they had finished calmly lifted himself up and snapped them all in two, remarking, "You thought yourselves very clever, didn't you?"

My little patient interrupts me about every three minutes to know if I have done yet, and tells me I am always writing, and when I go up to him and ask what he wants he says, "Tickle me," or something equally irrelevant. He is so pretty, has quite golden hair, in curls all over his head, and dark brown eyes and eyelashes. I have quite come to the conclusion that it is the clothes, and the enveloping dirt, that spoil poor people's children, as when they are clean and in bed they look just like a lot of little cherubs.

I have a tracheotomy case now under my special charge. A baby, nine months old, fat, and good tempered. I take it for twelve hours—nine a.m. till nine p.m.; and the night nurse for twelve—from nine p.m. to nine a.m. Of course I am relieved for meals, off-duty time, etc. It is a very interesting case, but rather an anxious one. I have been asked by the matron to stay on. I should like to, very much, but don't know how to get over the home difficulties. If only some kind angel would come down from heaven sometimes, and just point a finger in the really right direction!

(*To be continued.*)

### One Reason Why.

WONDER is constantly expressed that women brought up among the refined surroundings of homes of ease should so willingly, even eagerly, turn to the wearying, and often hard, routine of a hospital nurse's life, and find there, apparently, an attraction and a charm which were wanting in the home. Many explanations of this have been brought forward. We are told that the hospital uniform is becoming—there can be no doubt of the fact. We are told that many girls prefer the excitement of hospital life to the dulness they find at home. This is true. We are told again that some girls wish to improve their social position, and find a stepping-stone to a higher level by becoming a hospital nurse. I have no reason to doubt it, but, granting all this, I still venture the theory that the magnet which draws so many women within hospital walls is the fact that here in a greater measure than elsewhere does a woman find a life which satisfies many of the cravings of her nature. The more womanly the woman, the more does the work bring her the reward of "satisfied desire," which is another word for happiness itself. On entering a ward we leave most of the artificialities and empty forms of the outside world behind. We stand in a world of realities, where we see life from a new and truer standpoint. The importance and proportion of things are all changed. We do not ask if a nurse is well connected, but whether she knows her work and does her duty conscientiously. It matters not whether our patient is a gentleman or a rag-picker; the question is to do for him all that it is possible for human skill to do.

To a girl who has been in the habit of wearily finding something to fill the day—some occupation which, probably beginning with self, has ended with self—the blessed change of finding that every act of her daily life can be the means of comforting the weary bodies and souls of men, is a source of refreshment and pleasure untold. It is a privilege not given to every woman to know that in her vocation she can "Quérir quelquefois soulager souvent consoler *toujours.*" Where else can a woman be a "parson without his dogma, and a doctor without his drugs"?

It is a life so full of rewards for every service done that one can only wonder at those nurses (of whom one has heard sadly too much lately) who, apparently forgetful of the proportion of things, think lightly of the privileges they enjoy in being permitted to serve an apprenticeship to what, I believe, is the highest of all callings for a woman, and turn aside to grumble at the petty trials they meet—small grievances which greater-hearted women would not stop to notice.

We do not hear a thankful word for their golden opportunities of ministering to others. We hear instead many discontented words that they have not the food which exactly suits their fastidious tastes.

Many thousand loyal nurses are going quietly their way to-day, and are never heard. A few noisy, incompetent nurses bustle to and fro, clamouring for their rights, and the public, hearing their voices and no others, join in the wail, and bemoan the hard fate of nurses. I, who have been through three years' training in one of the London hospitals not noted for its easy work, can speak only of the happy fate of nurses. I feel convinced that any woman who chooses nursing as a profession and devotes all her best energies to the work will find herself repaid over and over again for the demands made upon her strength of body and mind.

Professor Drummond, in his latest book, speaking of loving service says: "As memory scans the past, above and beyond all the transitory pleasures of life, there leap forward those supreme hours when you have been enabled to do kindnesses to those about you. These seem to be the things which alone of all one's life abide. Everything else in all our lives is transitory. Every other good is visionary." To a nurse, more than most women, come these opportunities of doing the acts most worth doing in this world, and here I think we find the true secret of the attraction of hospital life.

DAGMAR.

### Everybody's Opinion.

[*Correspondence on all subjects is invited, but we cannot in any way be responsible for the opinions expressed by our correspondents. No communications can be entertained if the name and address of the correspondent is not given, or unless one side of the paper only be written on.*]

### ANOTHER HEROINE.

MISS EMMA DURHAM writes: Every reader of THE HOSPITAL will be glad to have had a glimpse of Mrs. Dacre Craven's picture, and will feel the honours and decorations given to her by Germany as given to themselves. Her work in the Franco-German war was, indeed, a great one, and I have heard something of it through my sister, who went to Metz to nurse, and bring back to England, Miss Barclay, one of Mrs. Dacre Craven's overworked nurses. I now beg to bring before your readers the name of another indefatigable woman, "Sister" Ruth Ray, who has been incessantly engaged in hard nursing work since 1870, without being fortunate enough to receive any decoration or honour except the Zulu War medal. Subjoined is a list of work in which she has taken part: At Westminster Hospital, from 1870 to 1873; superintendent nurse, Colonial Hospital, Port of Spain, Trinidad, from 1874 to 1877; with Lady Strongford in the Turkish War, from March to October, 1878; with the "Stafford House South African Aid Committee," Zulu War, from June to November, 1879; with Lady Strangford in

Egypt, from September to December, 1882; is now, and has been for the last eight years, matron of a small-pox and scarlet fever hospital.

### THE CASE OF THE OLDER NURSES.

A "Nightingale Sister" writes: May I write a few lines in your interesting paper to my colleagues who are getting beyond the age wanted in regular hospital work? From time to time I have been pained at seeing letters from nurses of forty or upwards who are wanting work, and feeling sore because hospitals do not want them. If only these nurses would take private service in the capacity of head nurses in gentleman's families they would secure a good home, regular income, with a healthy life of comparative ease. Many young married ladies would, I am sure, be glad to secure the services of a trained nurse as head in her nursery, provided the nurse was willing to do the work required. Of course, those nurses who object to clean lamps and lavatories would certainly not do for such a post. A nurse who has worked hard as a probationer, and been taught to do thoroughly the so-called menial work, would be invaluable to a lady, and would be able to train the nurses under her well. I know several ladies who would be willing to give salaries varying from £30 to £50 a-year for such a nurse; and in this case forty years of age would not be against them, but rather in their favour.

[The writer has herself taken up the work she advocates.—Ed.]

### MALE NURSES.

The Secretary of the Hamilton Association writes: The statements made by "A Male Nurse" in your number for October 4th call for a few comments. Your correspondent says "one can read 'self' on the part of association." It is evident that he writes from imperfect information. No profit has yet accrued to the association, but a sum of more than £1,200 has been spent in establishing it. Should any profit ever result it will be spent on the institution, which is a "voluntary association"—not a commercial company founded to produce dividends. As to fines, they were introduced so that there should be some punishment milder than that of expulsion. No man is kept permanently at hospital work, and at only one of the several hospitals we supply by the day is the rate of pay as low as £1 a-week. The majority of our nurses appear well satisfied, and, at any rate, the applications we receive almost daily far outnumber the vacancies on our nursing staff. A copy of the rules is enclosed; also one of the last annual report.

### Notes and Queries.

To Correspondents.—1. Questions may be written on post-cards. 2. Advertisements in disguise are inadmissible. 3. In answering a query please quote the number. 4. If a private answer is desired, a stamped addressed envelope must be forwarded.

Notice.—We must really ask our correspondents to be more particular in enclosing name and address. Constantly we receive queries or letters with no name attached.

### Queries.

(2) *Buenos Ayres.*—Information wanted about the climate, and the General Hospital, by a nurse going out.

(3) *Convalescent Home.*—Wanted for a poor woman just recovered from rheumatic fever. Must be free.—*Marjorie.*

(4) *Nettle Rash.*—What is the cause of nettle rash, and how long its duration?—*Nurse H.*

### Answers.

(3) *Convalescent Home.*—Try Mrs. Kitto's Home, South Park, Reigate; or Mrs. Gladstone's Home, Woodford, Essex.

*A. W.*—Thanks for the notes from lectures, but they are too similar to some already given to be of any use to us. Send us news, and we shall be much more grateful.

*Probationers.*—All ladies desiring to know the terms under which probationers are received for training at the different hospitals are requested to see the *Hospital Annual.*

*Paying Probationers.*—J. S. Ferrand, Detroit, should get the *Hospital Annual*, price 2s. 6d., to be had at our office. He will there find full particulars of the charges made for training by London and other hospitals in this country; the time required in the hospital for training; and the steps to be taken before certificate or diploma is granted.

### Appointments.

Richmond Hospital.—Miss Rachel Foley, who trained at the London Hospital, and has worked as head nurse at the Richmond Hospital for eight years, has been appointed Matron of that institution. The appointment has given great satisfaction in the neighbourhood, where Miss Foley is known and loved, though great regret is felt that Mrs. Liddall should resign.

Mill Lane Hospital.—Miss A. M. Whitfield, who trained at Addenbrookes under Miss Fisher, was appointed matron of this hospital at Sunderland on October 3rd. Miss Whitfield was for over two years housekeeper at the Northern Hospital, Liverpool, and has lately acted as matron of the Great Yarmouth Hospital.

Leeds Infirmary.—Miss Katherine Rapson, late sister of the Northumberland floor at the Seamen's Hospital, Greenwich, has been appointed assistant superintendent at the Leed's General Infirmary. Miss Rapson trained at Westminster. There were more than seventy candidates, and her election does her infinite credit. We hope she will be both successful and happy in her new sphere of work.

### Vacancies.

The following vacancies are announced:

**Nurses.**

Aberdeen Fever Hospital; £26.

Albert Edward Infirmary, Wigan; £25.

Bolton Fever Hospital; £25.

Blackheath and Richmond Institutions.

Bath Home.

Bournemouth Institution; £25.

Bristol Hospital for Children and Women.

Bromley Institution.

Burton-on-Trent Intitution; £25.

Chelsea Infirmary; £[illegible]8.

Coventry Hospital.

Cambridge Home.

Dewsbury Infirmary; £24.

Epsom Cottage Hospital; £20.

General Institute, Covent Garden.

Grosvenor Hospital, Westminster.

Hampshire Institute Southampton.

Hanover Institute, W.; £25.

Huddersfield Home.

Hull Home.

Hereford Infirmary; £20.

Homes for Aged Mariners, Cheshire; £25.

Hampstead Home Hospital.

Jersey Institute, £25.

Leicester Fever Hospital; £25.

Norwich Home.

Nottingham and Notts Institution; £25.

Ophthalmic Hospital, Moorfields.

Rochdale Infirmary; £22.

Royal Albert Hospital, Devonport; £25.

Southport Infirmary; £26.

Shoreditch Infirmary; £20 (temp., £22; assist., £17).

Suffolk General Hospital; £25 (night, £20).

St. Pancras Infirmary, Leavesden; £20.

Salford Infirmary, Eccles; £25.

Stamford Infirmary.

Smithston Hospital, Greenock; £25.

Sunderland Fever Hospital; £30.

Woolwich and Plumstead Cottage Hospital; £22.

Wilson Institution, Wimpole Street, W.; £30.

Workhouse Infirmary Association.

West Bromwich Hospital (night); £25.

**Probationers.**

Becket Hospital, Barnsley.

Haydock Cottage Hospital, Lancs.

Leeds Institution; £14.

Newport (Mon.) Infirmary.

Workhouse Infirmary Association

Weston-super-Mare Hospital.

Weymouth Hospital.

**Sister.**

Bolton Infirmary.

**District Nurse.**

St. Luke's Parish, Bromley Common.

### Amusements and Relaxation.

**N.B.-THIRD QUARTERLY WORD COMPETITION**

**Commenced Oct. 4, 1890; ends Dec. 27, 1890.**

Three prizes of 15s., 10s., 5s., will be given for the largest number of words derived from the words set for dissection.

The word for dissection for this, the FOURTH week of the quarter, being "PHYSICIAN."

| Names. | Oct. 16th. | Totals. |
|---|---|---|
| Jenny Wren | 46 | 101 |
| Tinie | — | 55 |
| Agamemnon | 43 | 98 |
| Patience | 43 | 98 |
| Ecla | 44 | 97 |
| Lightowlers | 4[illegible] | 95 |
| Rouge | 37 | 89 |
| Wyamaris | 36 | 81 |
| Qu'appelle | 36 | 81 |
| Nosam | 38 | 83 |
| Nurse Hilda | — | 44 |
| Lady Betty | 40 | 83 |
| Grenelle | — | 43 |
| Daisy | 36 | 76 |
| H. A. S. | 30 | 69 |
| A. B. C. | 28 | 66 |

| Names. | Oct. 16th. | Totals. |
|---|---|---|
| Liz | 30 | [illegible]8 |
| Checkmate | 43 | 76 |
| Silver King | 43 | 76 |
| S. Anthony | [illegible]3 | 76 |
| Quackah | 30 | [illegible]1 |
| Reynard | 32 | 62 |
| Sally | — | 27 |
| Success | 37 | 61 |
| Caledonia | 26 | 52 |
| Nurse Emma | 24 | 49 |
| Hazel | — | 20 |
| Pallas | 29 | 48 |
| Puss | — | 15 |
| Shakespear | 25 | 49 |
| Melita | 36 | 84 |

**Notice to Correspondents.**

Oct. 9th.—Melita, 48; Shakespear, 24.

